# *Aspergillus fumigatus* cytochrome c impacts conidial survival during sterilizing immunity

**DOI:** 10.1128/msphere.00305-23

**Published:** 2023-10-12

**Authors:** Matthew R. James, Mariano A. Aufiero, Elisa M. Vesely, Sourabh Dhingra, Ko-Wei Liu, Tobias M. Hohl, Robert A. Cramer

**Affiliations:** 1 Department of Microbiology and Immunology, Geisel School of Medicine, Dartmouth, Hanover, New Hampshire, USA; 2 Louis V Gerstner Jr. Graduate School of Biomedical Sciences, Sloan Kettering Institute, Memorial Sloan Kettering Cancer Center, New York, New York, USA; 3 Infectious Disease Service, Department of Medicine, Memorial Hospital, New York, New York, USA; 4 Human Oncology and Pathogenesis Program, Memorial Sloan Kettering Cancer Center, New York, New York, USA; University of Georgia, Athens, Georgia, USA

**Keywords:** *Aspergillus fumigatus*, reactive oxygen species, cytochrome c, sterilizing immunity, fungal virulence

## Abstract

Invasive pulmonary aspergillosis (IPA) is a life-threatening infection caused by species in the ubiquitous fungal genus *Aspergillus*. While leukocyte-generated reactive oxygen species (ROS) are critical for the clearance of fungal conidia from the lung and resistance to IPA, the processes that govern ROS-dependent fungal cell death remain poorly defined. Using a flow cytometric approach that monitors two independent cell death markers, an endogenous histone H2A:mRFP nuclear integrity reporter and SYTOX Blue (live/dead) stain, we observed that loss of *Aspergillus fumigatus* cytochrome c (*cycA*) results in reduced susceptibility to cell death from hydrogen peroxide (H_2_O_2_) treatment. Consistent with these observations *in vitro*, loss of *cycA* confers resistance to both NADPH oxidase-dependent and -independent killing by host leukocytes. Fungal ROS resistance is partly mediated by Bir1, a homolog to survivin in humans, as Bir1 overexpression results in decreased ROS-induced conidial cell death and reduced killing by innate immune cells *in vivo*. We further report that overexpression of the Bir1 N-terminal BIR domain in *A. fumigatus* conidia results in altered expression of metabolic genes that functionally converge on mitochondrial function and cytochrome c (*cycA*) activity. Together, these studies demonstrate that *cycA* in *A. fumigatus* contributes to cell death responses that are induced by exogenous H_2_O_2_ and by host leukocytes.

**IMPORTANCE:**

*Aspergillus fumigatus* can cause a life-threatening infection known as invasive pulmonary aspergillosis (IPA), which is marked by fungus-attributable mortality rates of 20%–30%. Individuals at risk for IPA harbor genetic mutations or incur pharmacologic defects that impair myeloid cell numbers and/or function, exemplified by bone marrow transplant recipients, patients that receive corticosteroid therapy, or patients with chronic granulomatous disease (CGD). However, treatments for *Aspergillus* infections remain limited, and resistance to the few existing drug classes is emerging. Recently, the World Health Organization classified *A. fumigatus* as a critical priority fungal pathogen. Our cell death research identifies an important aspect of fungal biology that impacts susceptibility to leukocyte killing. Furthering our understanding of mechanisms that mediate the outcome of fungal-leukocyte interactions will increase our understanding of both the underlying fungal biology governing cell death and innate immune evasion strategies utilized during mammalian infection pathogenesis. Consequently, our studies are a critical step toward leveraging these mechanisms for novel therapeutic advances.

## INTRODUCTION

*Aspergillus fumigatus* is a saprophytic fungus ubiquitous in the environment. This mold can cause a life-threatening lung infection known as invasive pulmonary aspergillosis (IPA) (1). IPA manifests due to defects in innate immune cell numbers and/or function that results in the failure to clear inhaled fungal conidia prior to germination and hyphal tissue invasion (2–4). Notably, individuals with chronic granulomatous disease (CGD) have impaired phagocyte NADPH oxidase function, and neutrophils from these individuals mount a defective respiratory burst against various microbial pathogens (5, 6). The IPA predisposition of individuals with CGD (and the susceptibility of CGD mice to *Aspergillus* challenge) indicate that products of host NADPH oxidase are required for *A. fumigatus* clearance from the lung (7, 8). While it is known that host-generated reactive oxygen species (ROS) are critical for sterilizing immunity in the lung, and conidia that undergo leukocyte mediated cell death display markers of regulated cell death (9), mechanisms that mediate fungal cell death from host–generated ROS remain ill-defined.

Cytochrome c is a key regulator of apoptosis in metazoa. Its release from the mitochondria under cell death inducing conditions, such as ROS exposure, promotes APAF-1-dependent activation of caspases (10, 11). Caspases are a family of cysteine proteases with specificity to aspartic acid residues that promote cell death via the controlled degradation of cellular proteins during apoptosis (12–14). Caspases are regulated by inhibitor of apoptosis proteins (IAPs) that directly inhibit caspase activity via a conserved baculovirus inhibitor of apoptosis repeat (BIR) domain (15–17). It was previously observed that loss of cytochrome C in *A. fumigatus* (encoded by the *cycA* gene) results in a strain capable of long-term persistence in the murine lung, resistance to murine macrophage mediated killing *in vitro,* and resistance to the superoxide generator menadione (18). Additionally, overexpression of Bir1, a homolog of the human IAP survivin, resulted in reduced fungal susceptibility to both exogenous ROS and *in vivo* leukocyte killing (9). While *A. fumigatus* contains these two genes canonically associated with caspase mediated cell death mechanisms in the metazoa, fungi do not encode caspases and questions remain regarding the role of fungal cytochrome c and survivin homologs in fungal cell death mechanisms.

In this study, we further explore the impact of cytochrome c on *A. fumigatus* susceptibility to reactive oxygen species and host leukocytes. Using a flow cytometry approach that monitors two independent cell death markers over time, we observe that loss of *cycA* confers reduced susceptibility to hydrogen peroxide (H_2_O_2_)–induced conidial cell death. Furthermore, by using the **FL**uorescent **A**spergillus **RE**porter (FLARE) method (7), we observe that loss of *cycA* impacts infection in an immunocompetent murine infection model and confers reduced susceptibility to both NADPH oxidase-dependent and -independent killing by lung neutrophils. We further report that the overexpression of the Bir1 N-terminal BIR domain results in altered transcript levels of a subset of genes that converge on mitochondrial and cytochrome c function. These results further our understanding of host-leukocyte mediated fungal gene dependent cell death in the setting of sterilizing immunity and open new questions about the role of fungal mitochondrial integrity and/or function in these processes.

## RESULTS

### *CycA* amino acid sequence annotation and conservation

**Fig 1:**
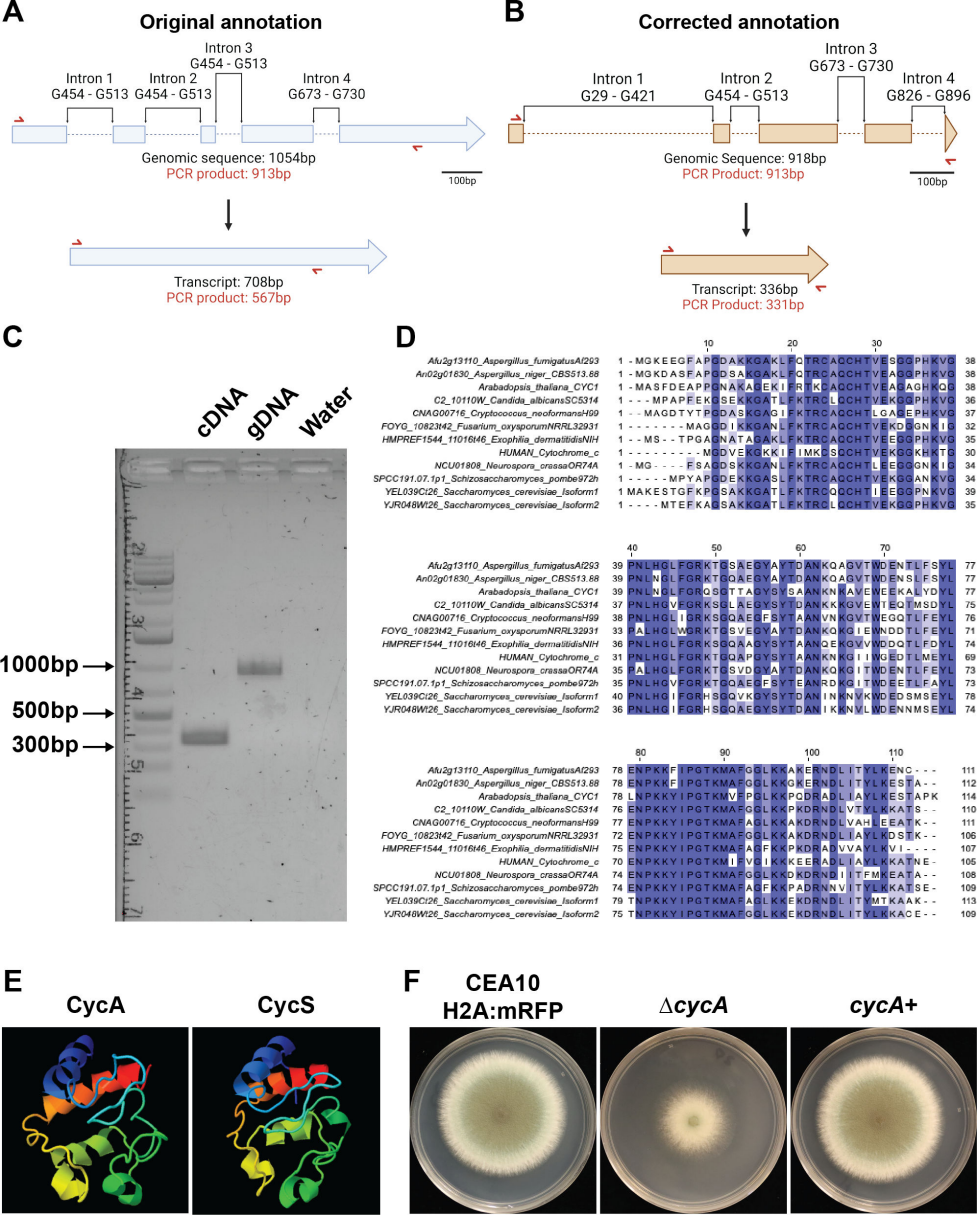
Cytochrome c sequence annotation in *A. fumigatus*. (**A**) Diagram of original annotation of *A. fumigatus cycA*. Top: diagram of genomic sequence including introns. Bottom: diagram of transcript size. Red arrows indicate the location of primers designed to test transcript size. (**B**) Diagram of corrected *cycA* annotation. Top: diagram of genomic sequence including introns. Bottom: diagram of corrected transcript. Red arrows indicate the location of primers designed to test transcript size. (**C**) Agarose gel displaying PCR products of primers (red arrows in Fig. 1A and B) for cDNA and gDNA. (**D**) Alignment of *A. fumigatus* cytochrome c to the sequences of other common organisms. (**E**) Phyre2 protein structure prediction of *A. fumigatus* cytochrome c protein (CycA) compared to human cytochrome c (CycS). (**F**) Representative images of the isogenic set used in this paper: CEA10 H2A:mRFP background strain, ∆*cycA*, and *cycA+* grown on glucose minimal media (GMM).

Cytochrome c is a highly conserved protein well known for its canonical function in the electron transport chain as an electron transporter between complex III and complex IV and as a mediator of metazoan apoptosis (10, 11, 19–21). Upon studying the *A. fumigatus* annotation of this gene in FungiDB, we noticed that the *cycA* genomic sequence was 1,054 base pairs (bp), resulting in a 708bp transcript, and subsequent 235 amino acid (aa) containing protein, which is nearly double that of known cytochrome c proteins in eukaryotes (Fig. 1A) (22). To resolve this unexpected finding, we obtained the AF293 reference nucleotide sequence from FungiDB and compared it to mRNA sequencing reads from references (23, 23). By analyzing sequencing reads in this data set, we identified four predicted introns: intron 1 (G29–G421), intron 2 (G454–G513), intron 3 (G673–G730), and intron 4 (G826–G896). The revised genomic sequence is 918bp in length, with a newly predicted TAA stop codon at T916. This results in a 336bp transcript, and subsequent 112 aa protein (Fig. 1B). To test whether our newly predicted annotation was correct, primers were designed (RAC7782 and RAC5421, Table S1) that would yield a 567bp cDNA product if the original annotation was correct, a 336bp cDNA product if our revised product was correct, and a 913bp band for the gDNA control (Fig. 1A and B, red arrow). From this PCR, we observed a cDNA product that corresponded to 336bp and a gDNA control band with the expected 913bp product, confirming that our newly predicted annotation is correct (Fig. 1C). Moreover, the corrected amino acid sequence displayed better coverage to cytochrome c proteins in other model organisms (Fig. 1D). When aligning the original CycA amino acid sequence, we observed only 41% coverage for *Aspergillus niger*, *Neurospora crassa*, *Arabidopsis thaliana*, and *Homo sapiens*. Using the revised amino acid sequence, we observed increased sequence similarity with other *Aspergillus* species orthologs; *A. niger* = 98%, *N. crassa* = 92%, *A. thaliana* = 89%, and *H. sapien* = 89%. Moreover, the revised amino acid sequence displayed high structural similarity to human *CycS* (Fig. 1E).

### Loss of *cycA* confers resistance to exogenous H_2_O_2_ treatment and alters cell death markers

**Fig 2:**
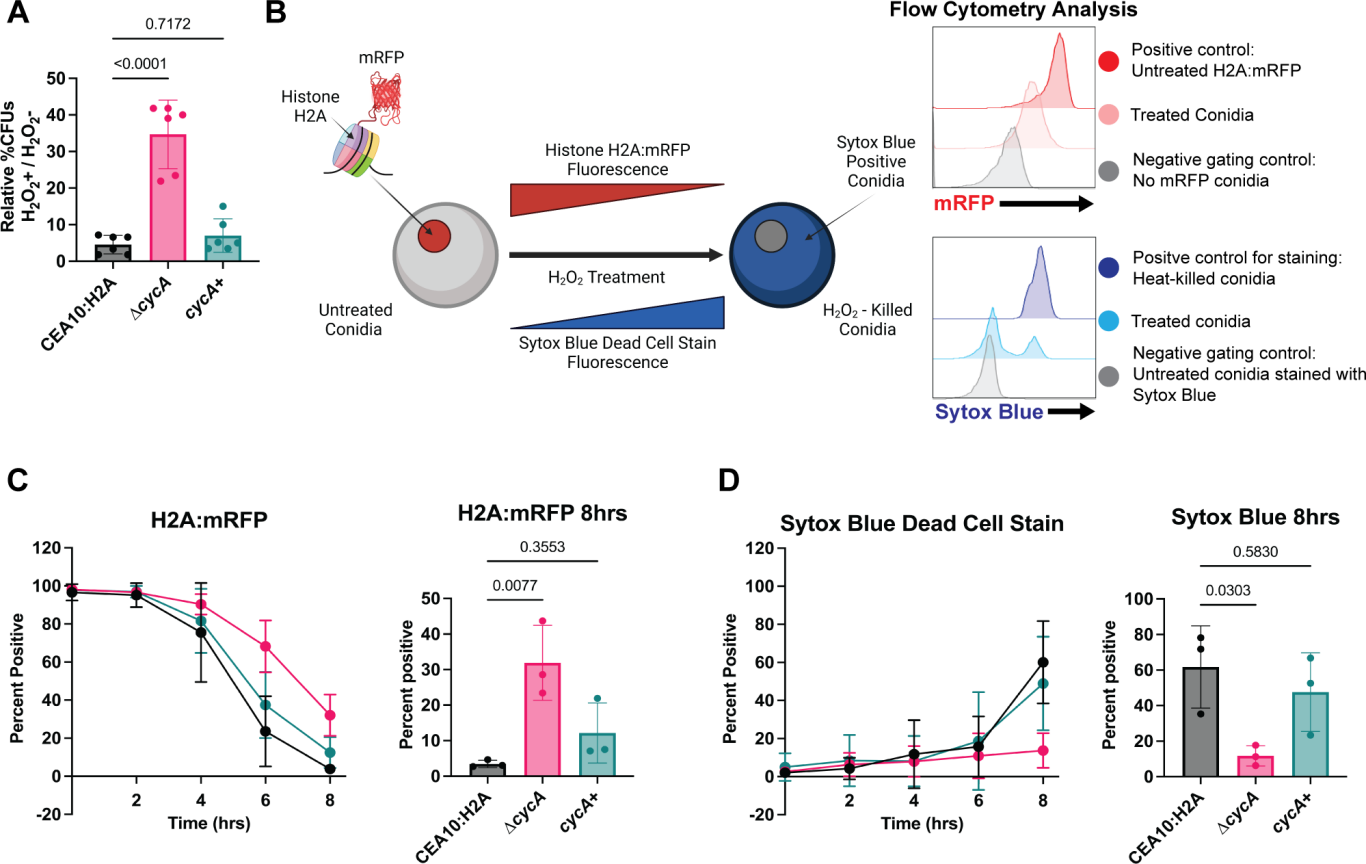
Loss of *cycA* alters multiple cell death markers after treatment with H_2_O_2_. (**A**) Normalized percent CFUs. Percent untreated = (5 mM treated CFUs/untreated CFUs). Data are representatives of two biological repetitions including three technical repetitions each. Statistical analysis: one-way ANOVA with Bartlett’s test. (**B**) Schematic of flow cytometry—based cell death assay experimental design. Over 8 hours treatment with 10 mM H_2_O_2_, H2A:mRFP conidia were analyzed by flow cytometry for mRFP fluorescence and SYTOX Blue fluorescence by flow cytometry. (**C**) Percent of histone H2A:mRFP positive conidia through H_2_O_2_ treatment (left) and quantification of histone H2A:mRFP positive cells at 8 hours (right). (**D**) Percent of SYTOX Blue positive conidia through H_2_O_2_ treatment (left) and quantification of SYTOX Blue positive cells at 8 hours (right). Data for C and D are representatives of three biological repetitions including 15,000 events each. Statistical analysis: one-way ANOVA with Bartlett’s test.

Previous work in our lab identified a role for *cycA* in the oxidative stress response, whereby loss of the *cycA* gene conferred resistance to the superoxide generator menadione (18). Since overexpression of full-length or the N-terminal domain of Bir1 was previously shown to confer resistance to H_2_O_2_ in swollen conidia (9), we tested the hypothesis that loss of *cycA* also confers resistance to H_2_O_2_ in swollen conidia. To analyze conidial cell death, we first generated a histone H2A:mRFP nuclear reporter strain that uses loss of mRFP fluorescence as a marker of conidia cell death (CEA10 H2A:mRFP) (9, 24, 25) (Fig. 2B). Using this strain, we generated the *cycA* null mutant CEA10 H2A:mRFP ∆*cycA* (referred to in this work as ∆*cycA*) by replacing the entire *cycA* gene with a hygromycin resistance cassette. We subsequently generated the reconstituted strain (*cycA*^+^) in the ∆*cycA* background (Fig. S1; Table S1). Consistent with previous work (18), we observed a marked growth defect in the ∆*cycA* strain as compared to the CEA10 H2A:mRFP background strain that was reconstituted by reinsertion of the *cycA* gene (Fig. 1F). Using this isogenic set, we observed that loss of *cycA* conferred an increase in viability after treatment with H_2_O_2_ as determined by the formation of CFUs (Fig. 2A). We next monitored two independent markers of conidia cell death over time; the endogenous H2A:mRFP signal and a SYTOX blue dead cell stain (26) (Fig. 2B). Every 2 hours, we stained a sample of H_2_O_2_–treated conidia, and analyzed these for H2A:mRFP and SYTOX blue fluorescence via flow cytometry. In the CEA10 H2A:mRFP strain, we observed that the majority of H2A:mRFP fluorescence signal loss occurred between 4 and 6 hours, consistent with previous observations (9). Between 6 and 8 hours, we observed a significant increase in SYTOX blue staining. For the ∆*cycA* strain, we observed a relative RFP fluorescence signal preservation during the treatment window, as judged by the presence of 31.9% mRFP^+^ cells compared to 3.48% (*P* = 0.0077) mRFP^+^ cells in the CEA10 H2A:mRFP background strain (Fig. 2C). In addition to preserved H2A:mRFP fluorescence, we observed that the ∆*cycA* strain had only 11.67% SYTOX blue^+^ conidia, while the CEA10 H2A:mRFP displayed 61.77% SYTOX blue^+^ conidia (*P* = 0.0303) (Fig. 2D). Taken together, these data suggest that loss of *cycA* reduces conidial susceptibility to H_2_O_2_–induced cell death.

### Loss of *cycA* confers resistance to conidial killing by host phagocytes

NADPH oxidase-derived ROS are essential for host defense against *A. fumigatus* (6). Consequently, we hypothesized that loss of *cycA* would render *A. fumigatus* resistant to phagocyte killing in the lung. We tested this hypothesis by challenging C57BL/6J mice with either 3 × 10^7^ WT, *∆cycA*, or *∆cycA+* conidia and analyzing lung phagocyte numbers and phagocyte uptake and killing of conidia at 36 hours post fungal challenge, using a fungal bioreporter, termed FLARE, as previously described (7). FLARE conidia encode RFP as a sensor of fungal cell viability and are labeled with Alexa Fluor 633 (AF633) as a tracer fluorophore. RFP^+^AF633^+^ leukocytes contain live fungal cells, while RFP^−^AF633^+^ leukocytes contain killed fungal cells. With this method, all AF633^+^ leukocytes represent leukocytes with phagocytosed fungal cells, irrespective of RFP fluorescence. The frequency of leukocytes with live fungal cells (AF633^+^RFP^+^) divided by the frequency of fungus-engaged leukocytes (all AF633^+^ leukocytes; fungus-engaged leukocytes) represents a measure of fungal cell killing.

**Fig 3:**
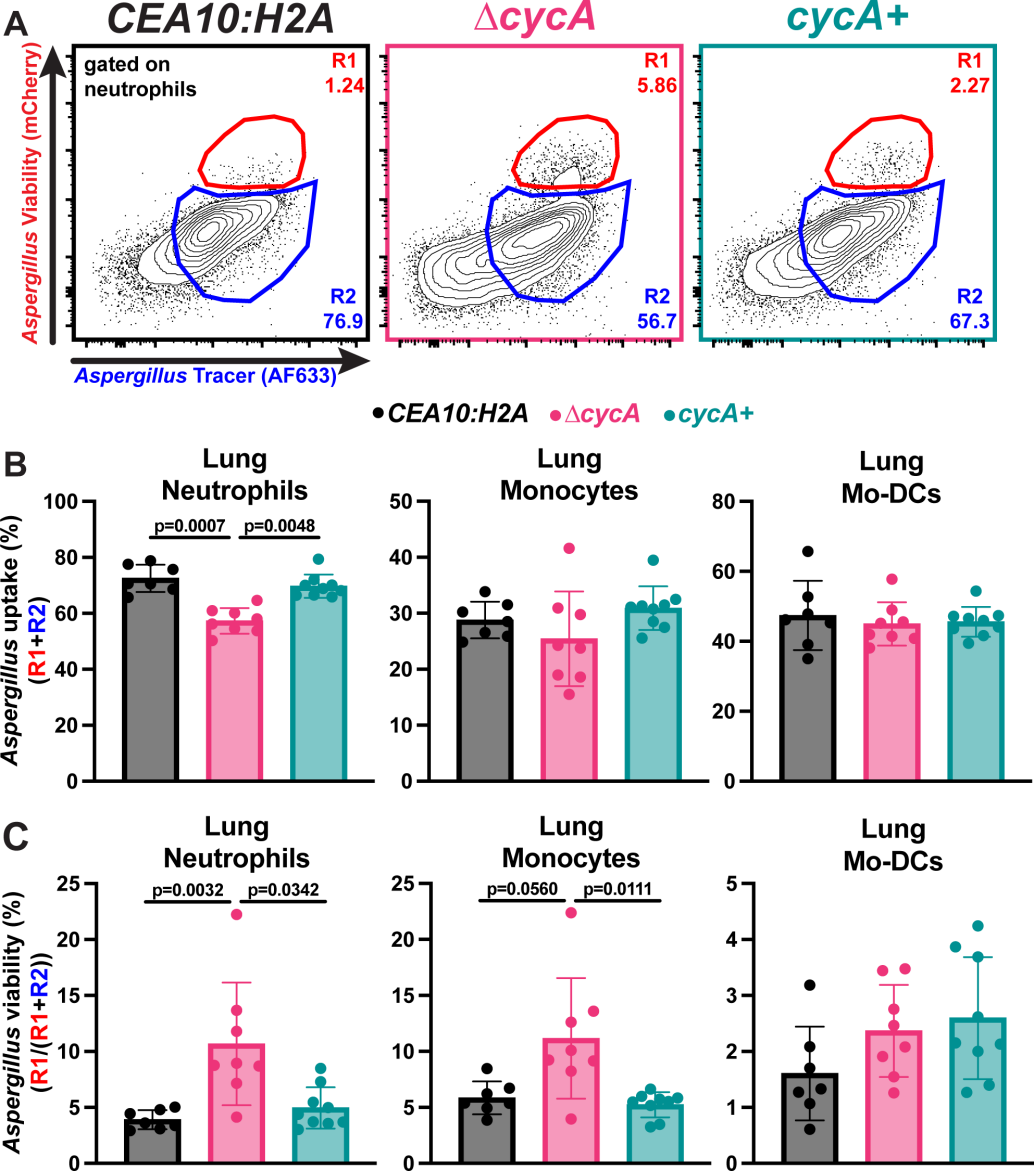
*Aspergillus fumigatus* ∆*cycA* is resistant to lung leukocyte killing. (**A**) Representative flow plots of lung neutrophil conidial uptake and killing, analyzed based on RFP and AF633 fluorescence, 36 hpi with 3 × 10^7^ AF633+ *CEA10:H2A* (black), *∆cycA* (magenta), or *∆cycA+* (teal) conidia. (**B and C**) The scatter plots indicate (**B**) conidial uptake (R1+R2) by and (**C**) conidial viability [R1/(R1+R2)] in indicated leukocyte subsets. (**B and C**) Dots represent individual mice and data are expressed as mean ± SEM. Statistical analysis: Kruskal–Wallis test with Dunn’s multiple comparisons test.

Compared to the *∆cycA+* strain, there were fewer neutrophils in the lungs of mice challenged with the *∆cycA* strain, but there was no difference in lung monocytes or monocyte-derived dendritic cells (Mo-DCs) (Fig. S2). We observed a slight decrease in neutrophil conidial uptake in mice challenged with the *∆cycA* strain. However, phagocytosis by monocytes or Mo-DCs was not affected by *cycA* loss (Fig. 3A and B). Strikingly, the fraction of fungus-engaged neutrophils and monocytes that contained live conidia was dramatically increased in mice challenged with the *∆cycA* strain compared to WT or *∆cycA+* (Fig. 3A and C). This result suggests that loss of *cycA* confers resistance to killing by lung myeloid phagocyte cells that are essential for anti-*Aspergillus* host defense.

**Fig 4:**
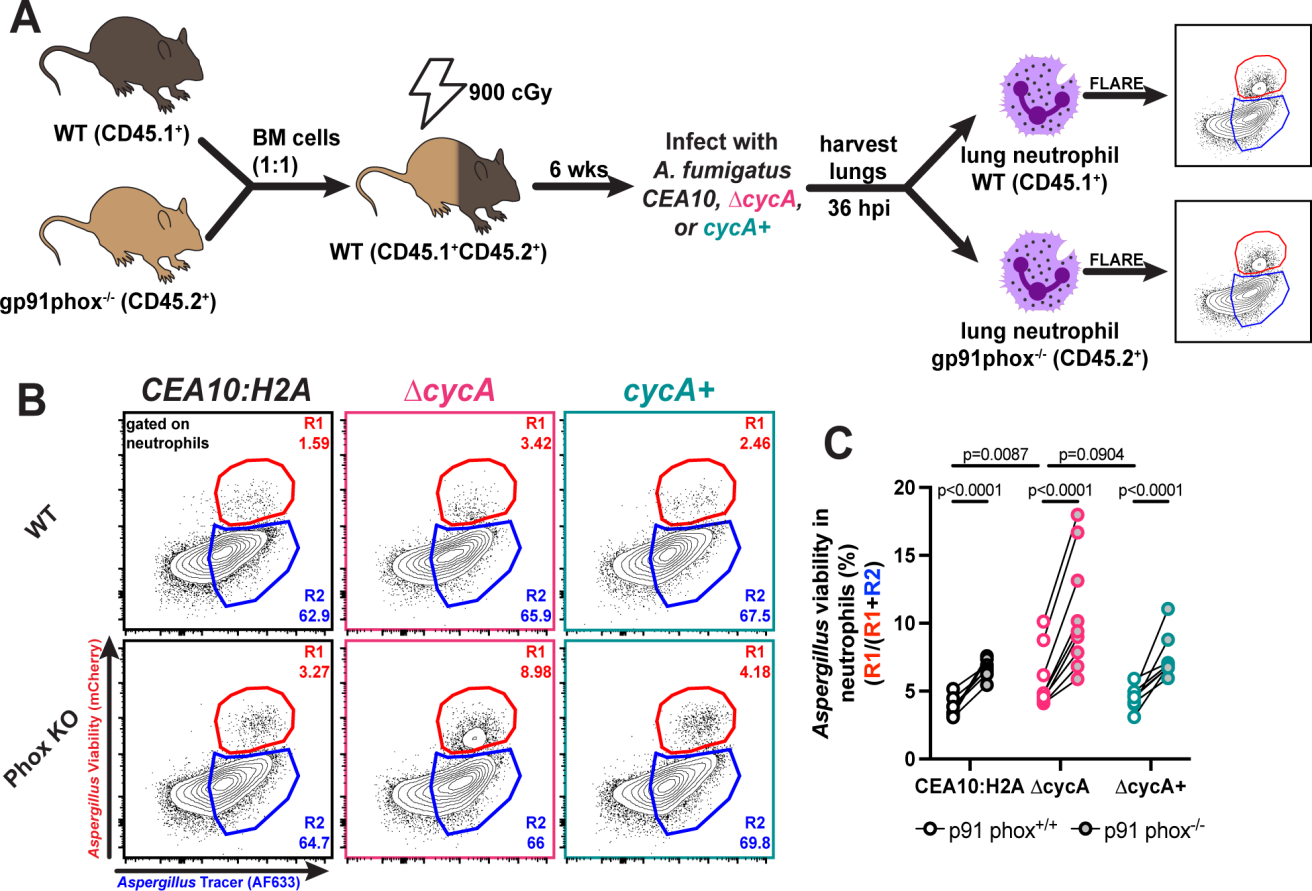
*∆cycA* conidia viability in NADPH oxidase deficient leukocytes. (**A**) Mice received a 1:1 mixture of p91phox^+/+^ and p91phox^−/−^ bone marrow, were rested for 6 weeks, and then challenged with 3 × 10^7^ AF633-labeled *CEA10:H2A* (black), *∆cycA* (magenta), or *∆cycA+* (teal). (**B**) Representative flow plots of lung neutrophil conidial uptake and killing from mixed bone marrow chimera mice, analyzed based on RFP and AF633 fluorescence, 36 hpi with 3 × 10^7^ AF633+ *CEA10:H2A* (black), *∆cycA* (magenta), or *∆cycA+* (teal) conidia. (**C**) The scatter plot indicates conidial viability in p91phox^+/+^ (white center) and p91phox^−/−^ (gray center) neutrophils. Dots represent individual mice with mean ± SEM indicated. Statistical analysis: Ordinary two-way ANOVA with main effects only and Sidak's multiple comparisons test, with a single pooled variance.

To test whether loss of *cycA* confers resistance to host NADPH oxidase-dependent killing, we generated bone marrow chimeric mice with equal ratios of gp91phox^+/+^ (an essential subunit of the NADPH oxidase complex) and gp91phox^−/−^ cells, and challenged these mice with WT, *∆cycA*, or *∆cycA+* strains (Fig. 4A). As expected, the fraction of viable WT and *∆cycA+* conidia increased in gp91phox^−/−^ neutrophils compared to gp91phox^+/+^ neutrophils, consistent with the known role of NADPH oxidase as a vital host mechanism for killing of *A. fumigatus* conidia in the lung (Fig. 4B and C). Surprisingly, the percentage of viable *∆cycA* conidia was also higher in gp91phox^−/−^ neutrophils compared to gp91phox^+/+^ neutrophils (Fig. 4B and C), indicating that NADPH oxidase-dependent mechanisms do not exclusively drive resistance to neutrophil killing conferred by loss of *∆cycA*. Our *in vitro* findings, which demonstrate enhanced resistance of *∆cycA* to chemically induced oxidative stress, combined with these *in vivo* results, suggest that the loss of *cycA* enables resistance to both oxidative and non-oxidative host killing mechanisms.

### RNA sequencing of Bir1^OE-N^ conidia shows altered mRNA transcript level differences that suggests altered metabolic function

**Fig 5:**
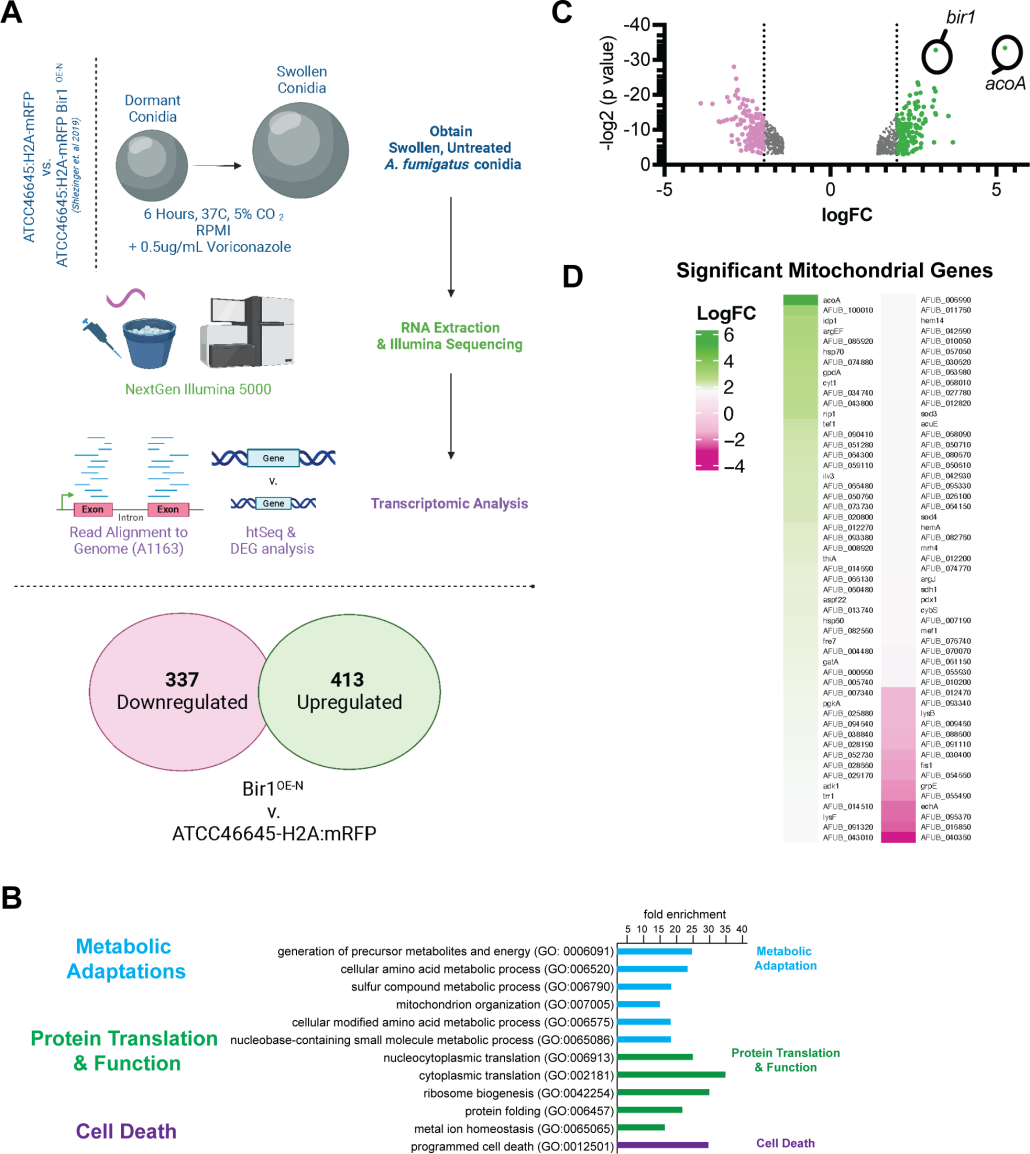
Oxidative stress-resistant Bir1^OE-N^ conidia show baseline transcript abundance variation. Transcriptional profiling of swollen conidia of strain ATCC46645-H2A:mRFP compared with an isogenic strain that features an over-expressed *bir1* (Bir1^OE-N^) gene (9). (**A**) Experimental set-up to determine gene expression in swollen conidia from both strains. (**B**) Differential gene expression comparing Bir1^OE-N^ to ATCC46645-H2A:mRFP, pink and green highlighting indicates genes with transcripts reduced and increased by more than logFC = 2, respectively. (**C**) Using FungiDB GO Term analysis, GO ”Mitochondria” gene transcripts were compared to Bir1^OE-N^ DEGs and visualized by heat map.

Recently, overexpression of the full-length (Bir1^OE^) or N-terminal Bir1 fragment that contains both BIR domains (Bir1^OE-N^) was observed to decrease *A. fumigatus* conidia susceptibility to exogenous H_2_O_2_ stress and result in increased fungal viability during leukocyte-fungal interactions in an immunocompetent murine infection model (9). These phenotypes are similar to the loss of *cycA* , and we next sought to more fully understand the genetic networks that dictate efficient oxidative stress induced killing of swollen fungal conidia. Therefore, we measured mRNA levels in Bir1^OE-N^ swollen conidia, previously described as N-BIR1^OX2^ (9), in comparison to the background strain (ATCC46645-H2A:mRFP) under basal conditions without exogenous ROS but including a sub-lethal dose of voriconazole to synchronize conidia in a swollen state (9) (Fig. 5A). To account for the use of voriconazole in the media, we tested whether the Bir1^OE-N^ resistance to H_2_O_2_ was dependent on voriconazole in the culture media. Using flow cytometry, we monitored loss of histone H2A fluorescence in Bir1^OE-N^ conidia as compared to the ATCC46645-H2A:mRFP background strain. From this, we observed that Bir1^OE-N^-dependent histone H2A:mRFP retention was not dependent on voriconazole in the media (Fig. S3).

RNA sequencing results indicated that transcripts of 413 genes were significantly increased, while 377 gene transcripts were decreased in mRNA abundance in the Bir1^OE-N^ strain compared to WT cells (twofold difference cutoff) (Fig. 5B). mRNA abundance of genes involved in ‘Ribosome biosynthesis’ and ‘Mitochondrial activity’ in Bir1^OE-N^ cells were elevated compared to the wild-type strain by Gene Ontology (GO) term analysis (Fig. 5C). The gene with the largest fold increase in mRNA levels in Bir1^OE-N^ as compared to WT was *acoA*, which encodes for aconitate hydratase in *A. fumigatus* (AFUB_001810, increased 26.2-fold, indicated in Fig. 5C). As expected, transcript levels of *bir1* itself were significantly increased as previously reported, confirming the over-expression strain. Moreover, we observed increased mRNA levels of genes that encode other key components of the electron transport chain in Bir1^OE-N^. These included complex II succinate dehydrogenase (AFUB_041300, 2.16-fold increased), complex III mitochondrial cytochrome b-c1 (AFUB_058230, 6.5-fold increased), mitochondrial cytochrome b-c1 (AFUB_002450, increased 6.8-fold), NADH-quinone oxidoreductase (AFUB_006990, 2.7-fold increased), NADH-ubiquinone oxidoreductase (AFUB_068090, 2.46-fold increased), and fumarate reductase (AFUB_082020, 4.5-fold increased) (Fig. 5D). These data suggest potential alterations in electron transport function in Bir1^OE-N^ cells.

We next examined mitochondrial genes with reduced mRNA levels in Bir1^OE-N^ vs wild-type swollen conidia and observed reductions in *echA* that encodes an Enoyl CoA-hydratase. A reduction in *echA* levels is consistent with a hypothesis that beta-oxidation may be suppressed in the utilized experimental conditions and that cells are not experiencing a “low glucose” state before RNA extraction (27). In addition, we observed a significant reduction in transcript levels of the gene responsible for regulation of mitochondrial fission*, fis1* (AFUB_028960, reduced threefold), suggesting that potential alterations in mitochondrial fusion and fission dynamics in Bir1N-OE cells may occur. In immortalized human cells, reductions in mitochondrial fission have been linked to both decreased apoptotic cell death and decreased leakage of cytochrome c from the mitochondria (28, 29). Interestingly, genes for secondary metabolite biosynthesis showed a reduced mRNA transcript abundance (e.g., polyketide F-9775B biosynthesis gene AFUB_033110, reduced 15.25-fold), which may indicate that Bir1^OE-N^ cells have a reduced biosynthesis of secondary metabolites under the conditions tested. Together, these data suggest that the leukocyte mediated cell death resistant Bir1^OE-N^ strain has altered transcriptional regulation of metabolic genes that functionally converge on cytochrome c activity and/or mitochondrial function.

## DISCUSSION

In this study, we utilized simultaneous tracking of two independent cell death markers to discover that loss of cytochrome c in *A. fumigatus* results in resistance to both chemically induced ROS *in vitro* and leukocyte-induced ROS in a murine model of infection. Interestingly, in the CEA10 H2A:mRFP strain, we observe that *in vitro* loss of H2A:mRFP fluorescence primarily occurs between 4 and 6 hours, while SYTOX Blue staining occurs mainly between 6 and 8 hours (Fig. 2C and D). These results suggest an order of events during conidial cell death in *A. fumigatus* where the loss of nuclear integrity measured as loss of mRFP fluorescence precedes loss of cell viability via membrane permeabilization during H_2_O_2_-induced conidial cell death. Loss of the *cycA* gene results in a reduction of these cell death markers, with overall more histone H2A:mRFP retention and less SYTOX blue dead cell staining. How these processes are temporally regulated is an intriguing question for future investigation, especially when put into the context of host-pathogen interactions of fungal conidia and the mammalian lung environment.

Consistent with our *in vitro* findings, we observed that at 36 hpi, the ∆*cycA* strain displayed increased viability in both lung neutrophils and lung monocytes (Fig. 3A through C). Moreover, in a mixed bone marrow experiment with both gp91phox^+/+^ and gp91phox^−/−^ leukocytes present, we observe a stark increase in viability in the ∆*cycA* strain in gp91phox^−/−^ neutrophils compared to gp91phox^+/+^ neutrophils (Fig. 4B and C). These data suggest that *cycA* confers resistance to host oxidative and non-oxidative forms of conidial killing. The decreased susceptibility of ∆*cycA* conidia to leukocyte killing may explain previous findings where it was reported that ∆*cycA* conidia are able to persist in the murine lung up to 29 days in immunocompromised models of infection despite a significant growth defect (18). The NADPH oxidase-independent factors contributing to conidial killing remain an area of active investigation, including further understanding the dynamics of the *A. fumigatus* cell death response to non-oxidative stressors *in vitro*.

The other *A. fumigatus* gene associated with leukocyte-mediated cell death and sterilizing immunity is Bir1. Overexpression of Bir1 or the N terminus of Bir1 confers phenotypes similar to loss of cytochrome c. To begin to determine if there is a mechanistic question between *bir1* and *cycA,* we utilized RNA-Seq analyses of the Bir1^OE-N^ strain. Interestingly, our transcriptome analyses of the *A. fumigatus* Bir1^OE-N^ stain reveal altered transcript levels of genes encoding proteins associated with the mitochondria and electron transport chain (Fig. 5). As an example, we observed changes in mRNA transcript abundance corresponding to genes critical for the incorporation of TCA cycle intermediates into the electron transport chain, most notably aconitate hydratase. Increased aconitate hydratase activity may result in increased flux through the TCA cycle resulting in increased incorporation of intermediates into the electron transport chain at Complex II (30, 31). These data suggest a model whereby swollen *A. fumigatus* Bir1^OE-N^ conidia may exhibit decreased susceptibility towards H_2_O_2_ stress due to increased electron flow through the electron transport chain from Complex II to Complex III and Complex VI (31). Electron transfer from Complex III to Complex IV occurs via cytochrome c, with the result facilitating downstream ATP synthase activity and providing cellular energy. Future studies aimed to understand the metabolic status of Bir1^OE-N^ conidia may observe increased mitochondrial respiration through ubiquinol-cytochrome b1c Complex III, resulting in mitochondrial membrane leakage, increased endogenous ROS, and increased ROS detoxification enzymes. Previous work (18), as well as our own findings on *cycA* and *bir1*, demonstrates support for a model where cytochrome c activity is critical for ROS sensitivity and that mitochondrial function and/or homeostasis is critical for survival when conidia are exposed to exogenous ROS and leukocytes.

Historically, research on regulated cell death (RCD) mechanisms has focused on metazoa (32–34), while RCD mechanisms outside of metazoa remain incompletely defined (35–37). In fungi, a complicating factor is the lack of many canonical cell death regulators described in metazoans such as caspases, caspase-interacting domains, and Bcl-2 family proteins, in fungal genomes. However, fungi encode homologs of mitochondrial-localized pro-apoptotic factors such as AifA, EndoG, HtrA2, cytochrome c, and homologs of gasdermin D and survivin (9, 25, 38–41). Aside from mammalian homologs, recent research suggests that cell death pathways unique to fungi exist. In *N. crassa*, the NLR-like protein PLP-1 functions in allorecognition by detecting SEC-9 from incompatible strains during heterokaryon incompatibility in germlings (42). Additionally, the *N. crassa* gasdermin-like protein, RCD-1, can promote cell death during heterokaryon incompatibility via interaction of two different alleles, *rcd-1–1* and *rcd-1–2*, which promotes the formation of pores on the plasma membrane (43). In *Saccharomyces cerevisiae*, AP-3 vesicle trafficking was implicated in cell death induced by acute heat stress, likely through function of the kinase Yck3 that leads to permeabilization of the vacuole (44). These results suggest that like metazoa, fungi may execute cell death processes in a regulated manner, and this may occur via conserved or fungal unique pathways.

Studies in both plant and human fungal pathogens have demonstrated that alteration of Bir1 transcript levels impacts susceptibility to cell death and virulence (9, 25). In the necrotrophic fungal plant pathogen, *Botrytis cinerea*, overexpression of Bir1, or its N-terminal domain, conferred reduced susceptibility to cell death induced by plant defenses and resulted in increased infection lesion size on *Arabidopsis thaliana* or *Phaselus vulgaris* leaves (25). In the human fungal pathogen, *A. fumigatus,* overexpression of Bir1, or its N-terminal domain reduced H2A:mRFP loss and TUNEL staining, and increased viability when swollen conidia were exposed to exogenous H_2_O_2_. Moreover, overexpression of Bir1 was protective against sterilizing immunity from host leukocytes and increased virulence in a murine model of fungal bronchopneumonia (9). In metazoa, both cytochrome c and survivin/Bir1 regulate the apoptotic pathway. Cytochrome c released from the mitochondria after apoptotic stimuli forms a complex with APAF-1, which catalyzes the activation of initiator and executioner caspases (10, 11). Conversely, one key role of survivin is to function as an inhibitor of apoptosis protein that directly inhibits both initiator and executioner caspases (15–17). Therefore, it is striking that in *A. fumigatus,* loss of CycA and overexpression of Bir1 are both protective under H_2_O_2_-induced cell death conditions independent of a known fungal caspase.

Putting our findings into this context, our data suggest an alternative model in which both CycA and Bir1 act within the same fungal death process. However, the reduced susceptibility to cell death observed in the ∆*cycA* strain could be a result of the defective ETC activity, thereby slowing down other cellular processes. As some forms of cell death, such as apoptosis, are dependent on ATP to proceed (45), our findings could shed light on a possible ATP dependent cell death process in fungi. Further investigation is required to determine if CycA and Bir1 participate in the same or different pathway(s) and if a cell death-specific protease is required for the altered H_2_O_2_ susceptibility of ∆*cycA* and Bir1^OE-N^ conidia. Understanding potential protein-protein interactions of both Bir1 and CycA during exposure to ROS may elucidate whether conidial cell death under these conditions is part of a conserved, regulated pathway or a consequence of an altered metabolic state. These areas of future investigation have the potential to provide novel insights into potential therapeutic targets for fungal infections and into the origin and execution of regulated cell death processes in pathogenic fungi.

## MATERIALS AND METHODS

### Strains and growth conditions

Mutant strains were generated in the CEA10/CBS144.89 strain (46). All strains were stored as conidia at −80°C in 25% glycerol. Culturing of strains was done using glucose minimal media (GMM). GMM media consists of 6 g/L NaNO_3_, 0.52 g/L KCl, 0.52 g/L MgSO_4_·7H_2_O, 1.52 g/L KH_2_PO_4_ monobasic, 2.2 mg/L ZnSO_4_·7H_2_O, 1.1 mg/L H_3_BO_3_, 0.5 mg/L MnCl_2_·4H_2_O, 0.5 mg/L FeSO_4_·7H_2_O, 0.16 mg/L CoCl_2_·5H_2_O, 0.16 mg/L CuSO_4_·5H_2_O, 0.11 mg/L (NH_4_)_6_Mo_7_O_24_·4H_2_O, 5 mg/L Na_4_EDTA, 10 g/L glucose. pH of media is adjusted to 6.5. To make solid GMM media, 1.5% agar (Difco granulated agar, BD Biosciences) was added to the above-described recipe. GMM media was autoclaved prior to use. For flow cytometry assays, liquid GMM was prepared as stated above, autoclaved, and subsequently filter sterilized using a 0.2 uM vacuum filter bottle system (Corning) to make optically clear GMM. To gather conidia, strains were cultured from −80°C stocks onto solid GMM and incubated for 3 days (4 days for the ∆*cycA* strain) at 37°C plus 5% CO_2_. Conidia were collected by addition of 0.01% Tween 80, followed by agitation with a cell scraper, and filtering through Miracloth.

### Culture conditions, RNA extraction, and RNA sequencing of Bir1^OE-N^ swollen conidia

Strains were plated onto LGMM and incubated for 72 hours at 37°C, 5% CO_2_ in the dark. After collection using 0.001% Tween 80 H_2_O, conidia were enumerated using a hemacytometer. In technical triplicate assays, 1 × 10^6^ ATCC46645-H2A:mRFP and ATCC46645-H2A:mRFP Bir1^OE-N^ conidia were inoculated in 100 mL of RPMI + 0.5 ug/mL Voriconazole (Sigma-Aldrich) and incubated at 50 RPM for 6 hours at 37°C. Fungal cells were collected by centrifugation and were flash frozen in liquid N_2_. Samples were then processed for RNA extraction after bead beating for 1 minute with 0.1 mm glass beads, followed by Trisure (Zymo Research) and chloroform extraction and purification through Qiagen columns (Qiagen RNAeasy Kit). RNA quantity and quality were estimated by Nanodrop and through gel electrophoresis before submission to the Dartmouth Genomics Core Facility. RNA was processed through Illumina NextSeq500 75 cycles after library preparation (Poly-A capture). Fasta files were further analyzed using FungiDB and EuPathDB Galaxy tools, mapping reads onto the closest related annotated genome, “A1163 Aspergillus fumigatus” (47). Resulting DeSeq2 from the comparison of “ATCC46645-H2A:mRFP” vs “Bir1-N” data were appended in Table S2. Differential expressed genes were visualized in Fig. 1B.

### Mutant strain generation

CEA10:H2A.X*^A. nidulans^*∷*ptrA* (CEA10 H2A:mRFP) was constructed in two steps. First, *gpdA*(p) was amplified from DNA isolated from strain *A. nidulans* A4 (Source:FGSC) using primers RAC2888 and RAC2799. Histone variant H2A.X (AN3468) was amplified (without stop codon) from A4 DNA (primers RAC4582 and RAC4583). mRFP fragment was amplified from plasmid pXDRFP4 (Source:FGSC) (primers RAC2600 and RAC4575), and terminator for *A. nidulans trpC* gene was amplified (primers RAC2536 and RAC2537). The four fragments were then fused together via PCR (primers RAC1981 and RAC4134), resulting in H2A.X first round fragment as described in reference (48). Secondly, we targeted integration of the H2A.X∷rfp to the intergenic locus between AFUB_023460 and AFUB_023470 on chromosome 2. For this, a left homology arm (primers RAC3873 and RAC3874) and right homology arm (primers RAC3875 and RAC3876) were amplified from CEA10 genomic DNA. Dominant selection marker gene *ptrA* conferring resistance to pyrithiamine hydrobromide was amplified from plasmid pSD51.1 (primers RAC2055 and RAC2056). The four fragments were then fused together via PCR (primers RAC3877 and RAC3878) as described earlier (48). After the construct generation, polyethylene- glycol-mediated transformation of protoplast was performed as described earlier (48). mRFP fluorescence was confirmed with FACS (fluorescence activated cell sorting) analysis. To generate CEA10 H2A:mRFP ∆*cycA* strain, PCR was used to amplify approximately 1 kb upstream (primers RAC5242 and RAC5243) and 1 kb downstream (primers RAC5244 and RAC5245) of the *cycA* gene. These upstream and downstream elements were fused to the hygromycin resistance gene (HygR) via PCR (primers RAC5246 and RAC 5247) to generate a knockout construct. This knockout construct was transformed into CEA10 H2A:mRFP protoplasts. Protoplasts were plated onto 15 mL of SMM (GMM plus 1.2M sorbitol) in 5 mL of SMM top agar (SMM + 0.7%agar). The following day a 5 mL layer of SMM top agar supplemented with hygromycin B (VWR) was added for a final concentration of 175 ug/mL hygromycin for the entire 25 mL SMM plate. To generate the *cycA* reconstituted strain (*cycA*+), the *cycA* loci, including ~1.3 kb upstream and 200 bp downstream of the open reading frame, was amplified out of the genome by PCR (RAC5246 and RAC5419). As the ∆*cycA* strain displays nearly no growth on media with glycerol as the only carbon source (18), this amplified PCR product was used as the full reconstitution construct and transformants were selected for on SMM agar with 1% glycerol instead of 1% glucose as the carbon source. For all transformations, protoplasts were generated treating germlings derived from liquid cultures grown at 28°C for 11 hours with lysing enzymes from *Trichoderma harzianum* (Sigma). Transformants were screened by PCR and confirmed with southern blot analysis.

Information regarding the Bir1^OE-N^ strain construction and confirmation of overexpression were previously published (9). Briefly, the overexpression construct was generated by cloning the first 1,545 bp of *A. fumigatus* Bir1 into pSK379. Transformants were selected on 2 µg/mL pyrithiamine.

### *cycA* sequence annotation correction and multiple sequence alignment

The *cycA* nucleotide and amino acid sequences for the reference strain AF293 (Afu2g13110) and CEA10 (AFUB_028740) were downloaded from fungiDB (47). Using the AF293 nucleotide sequence, we identified introns and a new stop codon based on reads identified in the (23) RNA seq data set (23). These newly determined introns were then verified with the combined AF293 RNA seq plot found on fungiDB. We then compared the nucleotide sequences of the updated AF293 introns and exons to that of CEA10 to determine any difference in amino acid sequences between the two. Protein structure modeling was done with Phyre2 (49) by comparing the updated AF293 *CycA* sequence to that of the human *CycS*. Protein sequences for orthologs of cytochrome c were obtained from UniProt or FungiDB and analyzed for similarity by multiple sequence alignment using ClustalW 2.1 and phylogeny.fr (50). Alignment was edited for clarity using JalViewJS (51). To confirm the corrected *cycA* transcript, primers RAC7782 and RAC5421 (Table S1) were used to amplify cDNA and DNA from the CEA10 H2A:mRFP strain using PrimeStar HS DNA polymerase (Takara).

### Cell death assays

2 × 10^6^ conidia/mL were swollen at 37°C for 4 hours in 10 mL cultures using optically clear LGMM. As the loss of *cycA* results in swelling defect, ∆*cycA* strains were swollen for an additional 2 hours (Fig. S4). After swelling, cultures were vortexed to resuspend and 1 mL of swollen culture was reserved for each strain as an untreated (0 hour) control. For 2, 4, 6, and 8 hours H_2_O_2_ treatment cultures, 1 mL of swollen conidia was aliquoted into a 5 mL screw cap centrifuge tube (VWR). To each 1 mL aliquot, 1 mL of 20 mM H_2_O_2_ in optically clear LGMM was added for a final concentration of 10^6^ conidia/mL and 10 mM H_2_O_2_ in 2 mL. H_2_O_2_-treated cultures were incubated in 37°C, and every 2 hours, a 2 mL treatment culture was stained with SYTOX blue dead cell stain (Invitrogen) at a concentration of 1 uM per manufacturer’s protocol and incubated at room temperature for 5 minutes. After staining, conidia were analyzed by flow cytometry. For flow cytometric analysis, 15,000 events were gathered for each strain at each timepoint. To process flow cytometry data, events were visualized on FSC-A vs FSC-H plots and gated for single events. Gating of events for mRFP fluorescence was determined by using conidia lacking mRFP as a negative control for gating and untreated (0 hour) H2A:mRFP conidia as a positive control. For SYTOX blue staining, a negative control for gating was used in which untreated conidia were stained with SYTOX blue dead cell stain. Based on these gates, the percent of mRFP positive and SYTOX blue positive singlets were derived for each treatment timepoint and compared to untreated (0 hr) fluorescence. All flow cytometry was conducted on a Cytoflex S cytometer (Beckman Coulter), and flow cytometry data analysis was done on FlowJo version 10.8.1.

### CFU assay

2 × 10^6^ conidia/mL were swollen at 37°C for 4 hours in optically clear LGMM. After swelling, conidia were treated with 5 mM H_2_O_2_ for 2.5 hours. After treatment, conidia were centrifuged to remove H_2_O_2_ media and resuspended in 0.01% Tween 80 in water. Conidia were enumerated using a hemacytometer and 100 conidia were plated in 5 mL of GMM top agar (GMM with 0.7% agar) overlaid (?) on 20 mL of GMM agar. Cultures were incubated at 37°C for 36 hours and colonies were counted. The number of colonies from H_2_O_2_-treated cultures was then compared to the strain specific untreated control groups.

### Mice

C57BL/6J mice (strain #: 000664) and p91phox^−/−^ mice (strain #: 002365) were purchased from The Jackson Laboratory. C57BL/6.SJL (WT CD45.1^+^) was purchased from Charles River Laboratories (strain # 00564) and crossed with C57BL/6 (CD45.2^+^) to generate CD45.1^+^CD45.2^+^ recipient mice for bone marrow chimeric mice.

### *In vivo* FLARE

To generate FLARE conidia, 7.5 × 10^8^ CEA10:H2A, *∆cycA*, and *∆cycA+* conidia were rotated in 0.5 µg/mL Sulfo-NHS-LC-Biotin (A39257; ThermoScientific) in 1 mL 50 mM carbonate buffer (pH 8.3) for 1 hour at RT, washed in 0.1 M Tris-HCl (pH 8), labeled with 0.02 mg/mL Streptavidin, Alexa Fluor 633 conjugate (S-21375; Molecular Probes) in 1 mL PBS for 30 minutes at RT, and resuspended in PBS and 0.025% Tween 20 for experimental use. For intratracheal challenge, mice were lightly anesthetized with isoflurane and immobilized in an upright position using rubber bands attached to a Plexiglas stand. The indicated number of conidia (typically 3 × 10^7^) in a volume of 0.05 mL PBS, 0.025% Tween 20 were delivered to the lung using a micropipette. To prepare single cell lung suspensions for flow cytometry, murine lungs were perfused and placed in a gentle MACS C tube and mechanically homogenized in 5 mL of PBS using a gentle MACS Octo Dissociator (Miltenyi Biotecc). Lung cell suspensions were lysed of RBCs, enumerated, and stained with fluorophore-conjugated antibodies prior to flow cytometric analysis on a Beckman Coulter Cytoflex LX and analyzed with FlowJo version 10.8.1. Neutrophils were identified as CD45^+^ CD11b^+^ Ly6G^+^ cells, inflammatory monocytes as CD45^+^ CD11b^+^ CD11c^−^ Ly6G^−^ Ly6C^hi^ cells, and Mo-DCs as CD45^+^ CD11b^+^ CD11c^+^ Ly6G^−^ Ly6C^hi^ MHC class II^+^ cells. Phagocytes that contain live conidia are RFP^+^ and AF633+ (R1) and phagocytes that contain dead conidia are RFP^-^ AF633^+^ (*R2*). Conidial phagocytosis was quantified as the sum of the fraction of a given phagocyte in the R1 gate and the fraction of a given phagocyte in the *R2* gate (R1+R2). To assess how effective phagocytes were at killing conidia, the fraction of viable conidia was calculated as R1/(R1+R2).

### Mixed bone marrow chimera

Lethally irradiated (900 cGy) F1 progeny (from a cross of C57BL/6 and C57BL/6.SJL strains) were reconstituted with a mixture of 1–2.5 × 10^6 ^CD45.1^+^ p91phox^+/+^ and 1–2.5 × 10^6^ CD45.2^+^ p91phox^−/−^ bone marrow cells, treated with enrofloxacin in the drinking water for 21 days to prevent bacterial infections, and rested for 6 weeks prior to use. Mice were challenged with 3 × 10^7^ WT, ∆*cycA*, or ∆*cycA*+ FLARE conidia and after 36 hours were analyzed by flow cytometry as described above.

## Data Availability

Raw and processed RNA sequencing data in this study are available at the GEO repository under accession number GSE233942. All diagrams were generated using BioRender.com. All data was processed using Prism GraphPad version 9.5.1.

## SUPPLEMENTAL MATERIAL

Fig. S1 msphere.00305-23-s0001.tif Strain construction.

Fig. S2 msphere.00305-23-s0002.tif Flow cytometry gating.

Fig. S3 msphere.00305-23-s0003.tif Voriconazole and DMSO control experiment.

Fig. S4 msphere.00305-23-s0004.tif Fungal flow cytometry gating for singlets.

Table S1 msphere.00305-23-s0005.xlsx Primers and strains.

Table S2 msphere.00305-23-s0006.xlsx GO term analysis.

ASM does not own the copyrights to Supplemental Material that may be linked to, or accessed through, an article. The authors have granted ASM a non-exclusive, world-wide license to publish the Supplemental Material files. Please contact the corresponding author directly for reuse.
