# Supplementary figures and images for: Aspergillus fumigatus cytochrome c impacts conidial survival during sterilizing immunity
Source: mSphere. 2023 Oct 12;8(6):e00305-23. doi: 10.1128/msphere.00305-23 (PMC10871163; doi:10.1128/msphere.00305-23)

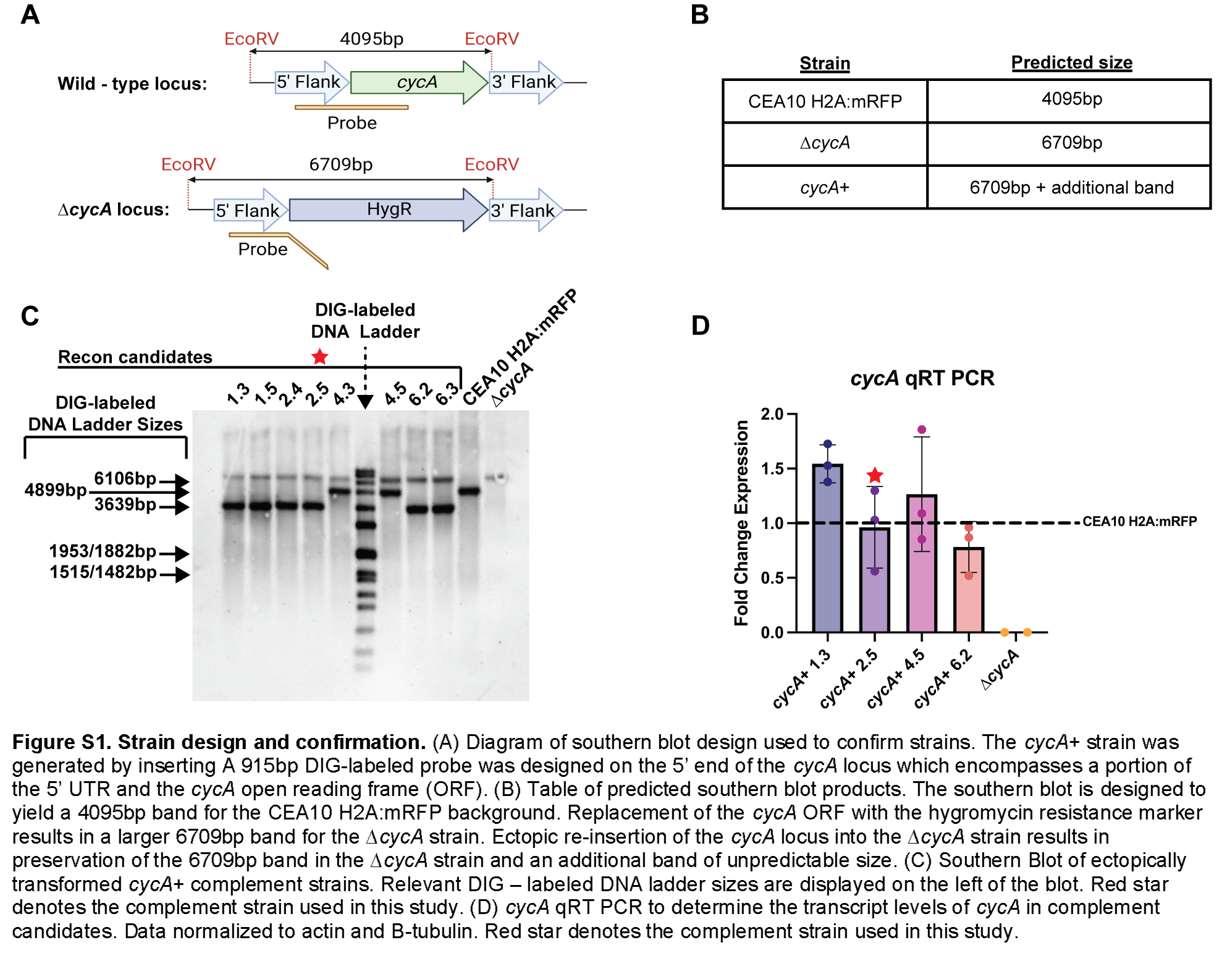

Supplement: Fig. S1 — Strain construction. [file msphere.00305-23-s0001.tif]

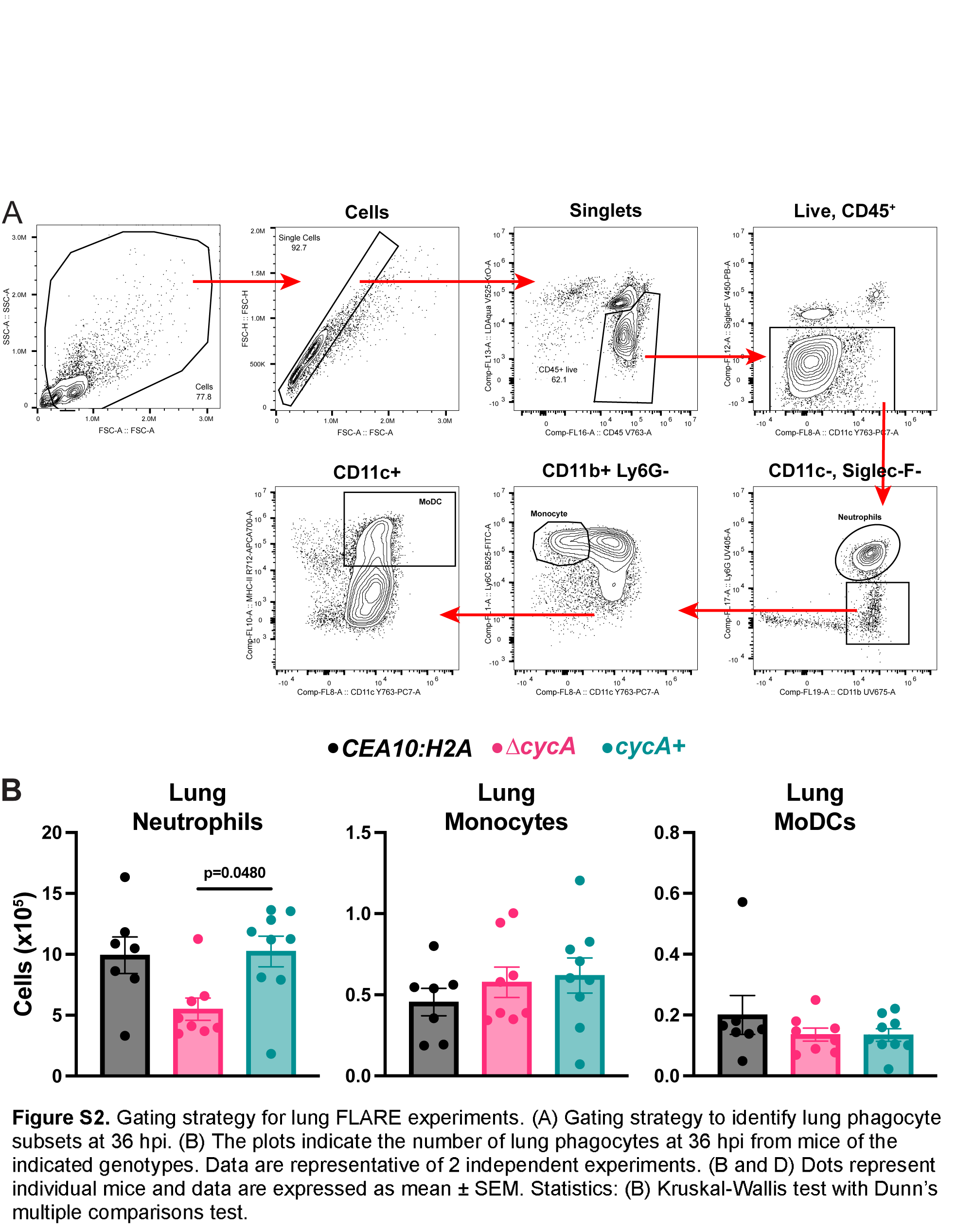

Supplement: Fig. S2 — Flow cytometry gating. [file msphere.00305-23-s0002.tif]

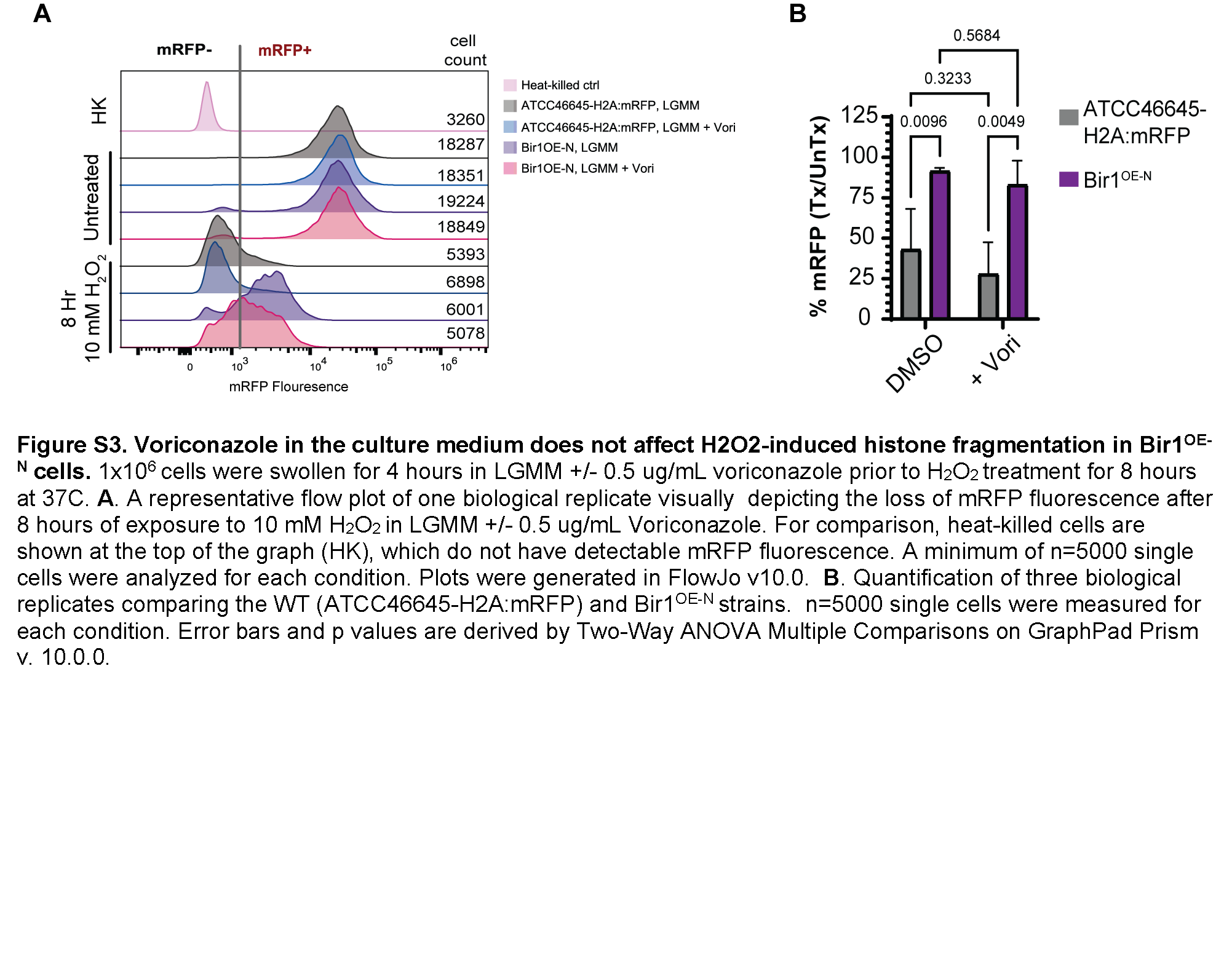

Supplement: Fig. S3 — Voriconazole and DMSO control experiment. [file msphere.00305-23-s0003.tif]

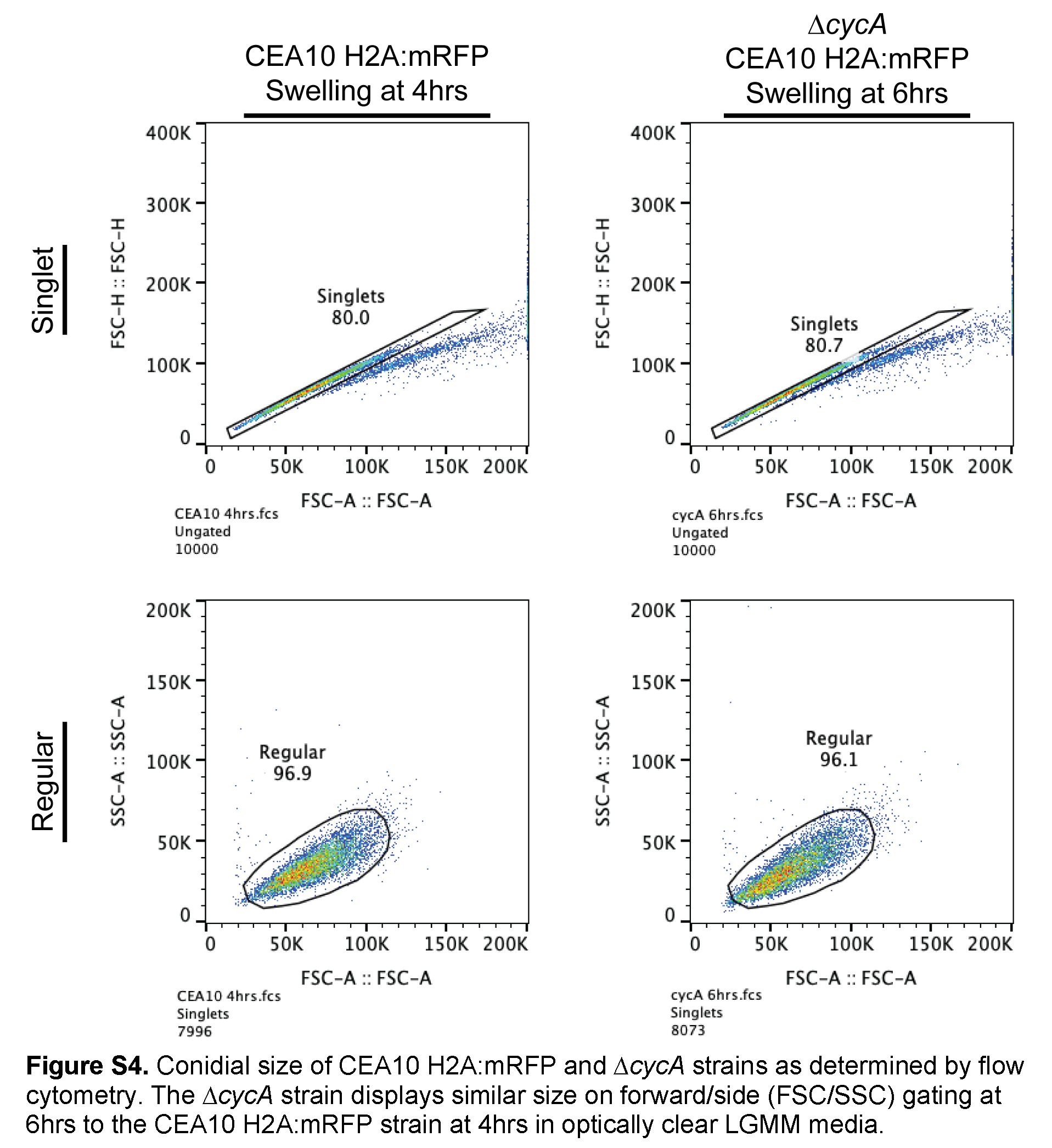

Supplement: Fig. S4 — Fungal flow cytometry gating for singlets. [file msphere.00305-23-s0004.tif]
